# Effect of hospice care on quality indicators of end-of-life care among patients with liver cancer: a national longitudinal population-based study in Taiwan 2000–2011

**DOI:** 10.1186/s12904-015-0036-9

**Published:** 2015-08-19

**Authors:** Yee-Hsin Kao, Jui-Kun Chiang

**Affiliations:** Department of Family Medicine, Tainan Municipal Hospital, Tainan, Taiwan; Department of Family Medicine, Buddhist Dalin Tzu Chi Hospital, 2 Minsheng Road, Dalin, Chiayi Taiwan

## Abstract

**Background:**

Quality of near end-of-life (EOL) care is typically evaluated using six accepted quality indicators (QIs). Research has yet to evaluate the quality of EOL care for liver cancer patients in Taiwan. We evaluated the effect of hospice care on the quality of EOL care for patients with advanced liver cancer.

**Methods:**

Using claims data obtained from the Taiwan National Health Insurance Research Database, we analyzed the QIs of EOL care for patients who died between 2000 and 2011. Logistic regression was performed to identify predictors for QIs of EOL care.

**Results:**

A total of 3092 adult patients died of liver cancer during the study period. The patients were divided into those who received hospice care for a period longer than 1 month (long-H group), shorter than 1 month (short-H group), and not at all (non-H group). There was no significant difference in survival probability among the three groups (*p* = 0.212). Compared with the non-H group, the long- and short-H groups exhibited a significantly lower risk of being admitted to an intensive care unit (ICU) (odds ratios [ORs] = 0.25 and 0.26, respectively, *p* < 0.001) and requiring cardiopulmonary resuscitation (CPR) during the final month of life (ORs = 0.21 and 0.09, respectively, *p* < 0.001). Compared with the non-H group, the short-H group had a higher risk of more than one emergency room (ER) visit, and more than one hospital admission (OR = 1.97, *p* = 0.003; and OR = 1.56, *p* = 0.001, respectively), but the long-H group did not differed significantly from the non-H group on these measures.

**Conclusions:**

Patients with liver cancer who received hospice care were less likely to be admitted to ICUs or require CPR compared with those who received no hospice care. A longer duration of hospice care was associated with reduced risks of more than one ER visit and more than one hospital admission. We conclude that EOL cancer care in Taiwan might be improved by implementing policies encouraging early hospice referral programs.

**Electronic supplementary material:**

The online version of this article (doi:10.1186/s12904-015-0036-9) contains supplementary material, which is available to authorized users.

## Background

Cancer, a leading cause of death worldwide, accounted for 8.2 million deaths in 2012 [1]. Although the diagnostic practices and treatments for cancers have improved, the mortality rate from this disease has not [2, 3]. In Taiwan, 43,665 (28.4 %) of the people who died in 2012 died from cancer [4]. Because of the high mortality rate, a complete cancer treatment program requires a consideration of the quality of near end-of-life (EOL) care.

Six quality indicators (QIs) of EOL cancer care have been accepted and validated: (1) receiving chemotherapy during the final 2 weeks of life, (2) having more than one emergency room (ER) visit, (3) being admitted to a hospital more than once, (4) receiving care in an intensive care unit (ICU) during the final month of life, (5) receiving cardiopulmonary resuscitation (CPR) during the final month of life, and (6) dying in an acute care hospital [5, 6]. These six QIs of EOL care have been adopted in the United States [5, 7], Canada [8], and Taiwan [9] and are considered indicators of aggressive care at EOL. Because aggressive care at EOL is inappropriate for the dying, these indicators can be used to evaluate the quality of hospice care programs.

Although hospice care is currently considered appropriate for terminally ill patients in Taiwan and has been increasing for more than 20 years, it remains underutilized. In Taiwan, the rate of patients with any type of cancer who received hospice care during their final year of life increased from 7.34 % in 2000 to 16.83 % in 2006 [10]; however, the overall prevalence remained low. Hospice patients can receive care, such as emergency room (ER) visits, hospital admission, and anticancer treatment, if they require symptom relief. In Taiwan, hospice care service models are delivered by hospital-based hospice care units, which provide both inpatient and home care services. To increase hospice care coverage, hospice shared care is a new care model that has been used since 2005 to treat advanced cancer inpatients admitted to non-hospice wards [11]. There are no independent hospices in the community in Taiwan. Among patients receiving hospice care, 12.4 % receive it at home and 87.6 % receive it in a hospital [9]. Patients diagnosed with advanced progressive cancers with a prognosis of approximately 6-month survival are eligible for hospice care. The assessment criteria applied in this study were in accordance with Ministry of Health regulations. The application of hospice care was assessed by the hospice care team. If patients with terminal illnesses require hospice services, they must be transferred to hospice care wards; many patients or their families express a wish for do-not-resuscitate orders.

In the United States and Canada, hospice care is mostly home based. In Taiwan, although hospice services can be delivered to patients in their homes, they are predominantly provided in hospital hospice wards. Both forms of hospice care are covered by Taiwan’s National Health Insurance (NHI) program; therefore, patients do not need to pay for this service. Previous studies have applied the care QIs to evaluate EOL care for patients with any cancer type [8, 12–14] or specifically lung [15], colorectal [16], and breast [17] cancer; however, the findings have been inconsistent. For example, Saito *et al.* reported improvements in all six QIs [15]. Dudgeon *et al.* reported a decrease in the percentage of multiple ER visits and multiple hospital admissions, but a nonsignificant decrease in the percentage of deaths in a hospital setting [12]. No study has evaluated EOL care for patients with terminal liver cancer, which is a leading cause of cancer death in Taiwan [4].

In this study, we used claims date from the Taiwan NHI Research Database (NHIRD) to evaluate the effect of hospice care on the QIs of EOL care for terminal liver cancer patients in Taiwan.

## Methods

### Data source and identification

Taiwan’s NHI program is a single-payer health insurance system implemented in March 1995 and had a high national coverage rate of 98.4 % in 2007 [18]. The Taiwan NHIRD contains original claims data of nearly all of Taiwan’s more than 23 million residents. In Taiwan, all cancer patients are designated as having a catastrophic illness. We first identified all patients with catastrophic illness designations in Taiwan’s Longitudinal Health Insurance Database 2000, a random and systematic sample of one million NHI beneficiaries from 2000 to 2011. We then used the claims data of these patients to identify patients with primary liver cancer, defined according to the International Classification of Diseases, Ninth Revision, Clinical Modification (ICD-9-CM) codes 155, 155.0, and 155.1. The patients were further subcategorized according to the presence of comorbid conditions, including hepatitis B virus (HBV) (ICD-9-CM codes 070.20–070.23, 070.30–070.33, and V0261) and hepatitis C virus (HCV) (ICD-9-CM codes 070.41, 070.44, 070.51, 070.54, and V0262). Other comorbid conditions were defined as in previous studies [19, 20]. Patients were excluded if they died at an age younger than 20 years or had no definite death date, no insurance claims during their final year of life, or inaccurate (e.g., their death date was earlier than their diagnosis date) or missing data.

### QIs of EOL cancer care in the final month of life

The literature review revealed six widely accepted QIs of cancer care in the final month of life [5, 9, 21, 22]; namely (1) receiving chemotherapy during the final 2 weeks of life, (2) having more than one ER visit during the final month of life, (3) being admitted to a hospital more than once during the final month of life, (4) being admitted to an ICU during the final month of life, (5) receiving CPR requiring intubation and mechanical ventilation during the final month of life, and (6) dying in acute care wards or hospice wards in the hospitals. Death in the hospital was defined as a date of discharge that was the same as the date of death [22]. Because chemotherapy is not commonly used to treat patients with liver cancer, the indicator of chemotherapy during the final 2 weeks of life was changed to anticancer therapies, including transarterial chemoembolization, hepatic arterial infusion chemotherapy, percutaneous ethanol injection, radiofrequency ablation, and radiotherapy in the final month of life. Sorafenib, the target therapy for hepatocellular carcinoma, was not included in this study because it was not introduced to Taiwan until 2010, the final year of our 10-year study period. These six QIs of EOL care have been adopted by researchers from the United States [5, 7], Canada [8], and Taiwan [9] and illustrate the quality of care for patients with cancers in the last month of life.

Hospice patients were defined as those with claims for hospice care at least once between diagnosis and death, whereas non-hospice patients (non-H group) were those without such claims. Hospice patients were further divided into two groups according to whether the length of hospice care was shorter (short-H group) or longer (long-H group) than 1 month. Patients in the short-H group received hospice care in the last month of life and patients in the long-H group received hospice care for longer than the last month of life. This classification was also convenient for the analysis. Length of hospice care was calculated from the date of first hospice service until death.

The patients were further subcategorized according to family socioeconomic status (SES) based on previous studies [23,24]. A family earning less than NT$20,000 (approximately US$571), NT$20,000–40,000 (US$571–1141), and more than NT$40,000 (US$1141) per month, as listed in the claims data, was defined as having a low, moderate, and high SES, respectively.

The Research Ethics Committee of Buddhist Dalin Tzu Chi Hospital, Taiwan, reviewed and approved the protocol of this study (No. B10301001). Because the NHIRD files contain only de-identified secondary data, the review board waived the requirement for informed consent.

### Statistical analysis

Mean ± standard deviation (SD) and frequency (proportions) were used to describe group characteristics. Categorical variables were analyzed using the chi-square test, and continuous variables were compared using the *t* test or the Wilcoxon rank-sum test, as appropriate. The Kaplan–Meier method was adopted for survival data analyses. Multiple logistic regression analysis with a stepwise variable selection procedure was conducted to compute adjusted odds ratios (ORs) and 95 % confidence intervals (CIs) for the association among the QIs of EOL cancer care. Generalized additive models were fitted to detect potential nonlinear effects of continuous Licensecovariates and identify an appropriate cutoff point for discretizing continuous predictors, such as age, during stepwise variable selection. Area under the curve (AUC) analysis for receiver operating characteristic curves was performed. We assumed that an AUC of ≥0.7 indicated an acceptable level of discrimination for the fitted logistic regression model. The goodness of fit of the logistic regression model was assessed using the Hosmer–Lemeshow test. Statistical analysis was performed using R, Version 2.15.1 (The R Foundation). A two-sided p < 0.05 was considered significant.

## Results

**Fig. 1:**
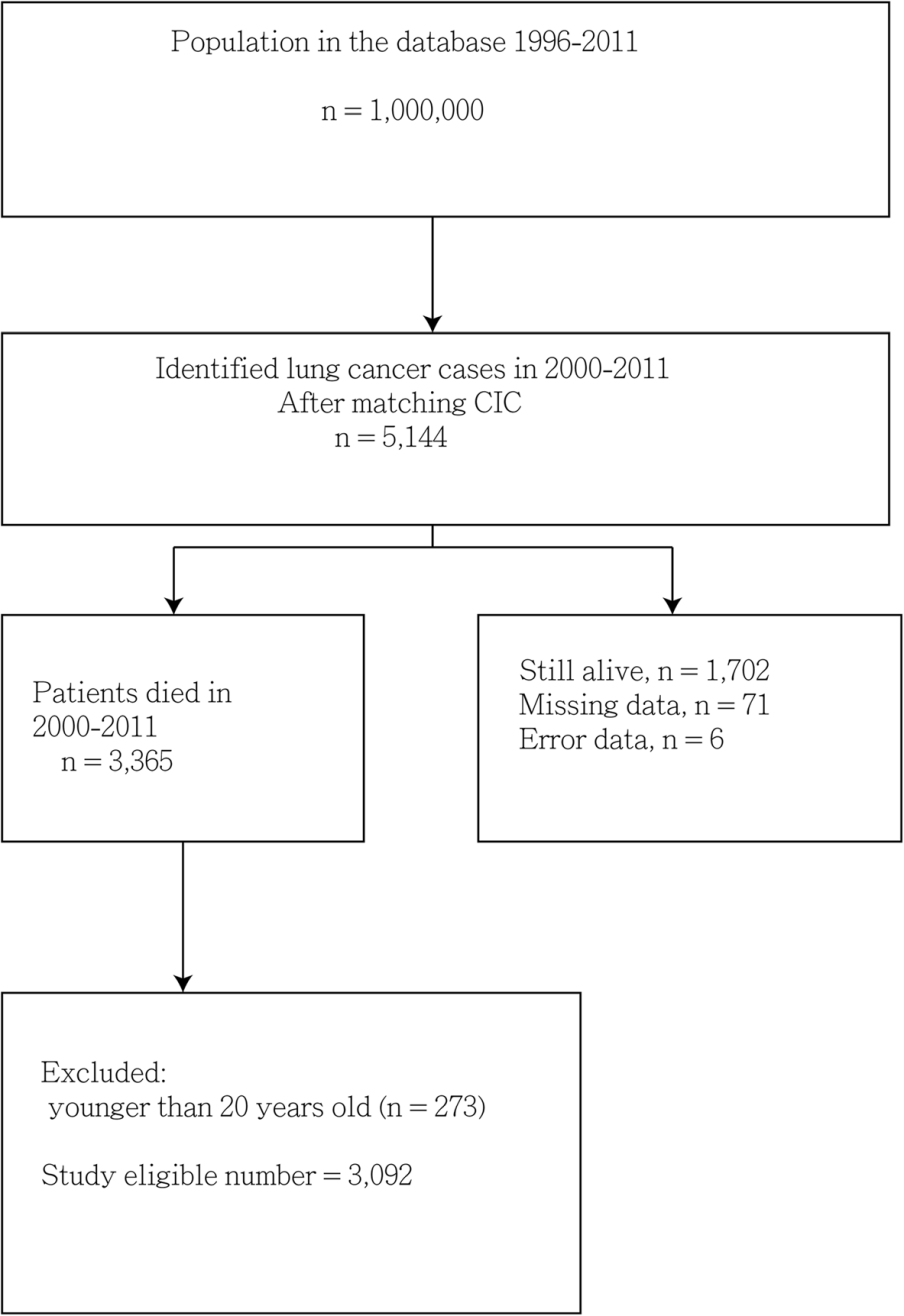
Study flow chart. Abbreviations: ICD-9-CM, International Classification of Diseases, Ninth Revision, Clinical Modification; CIC, catastrophic illness certificate

**Fig. 2:**
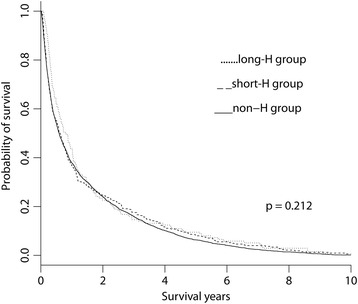
The Kaplan–Meier estimates of survival curves for advanced liver cancer patients stratified by hospice care. Patients were grouped according to those who received hospice care for longer than 1 month (long-H group), shorter than 1 month (short-H group), and not at all (non-H group)

After exclusion, we observed that 3092 adult patients (2216 men, 876 women; ratio = 2.5:1) died of liver cancer between 2000 and 2011. Figure 1 shows the enrollment flow chart. Of the patients, 137 (4.4 %) were in the long-H group, 325 (10.5 %) were in the short-H group, and 2630 (85.1 %) were in the non-H group. The mean ± SD survival probabilities (years) after diagnosis in the long-H, short-H, and non-H groups were 1.60 ± 0.17, 1.52 ± 0.11, and 1.40 ± 0.04 years, respectively. There was no significant difference in survival probability among the three groups (log-rank test p = 0.212) (Fig. 2). The mean ± SD (median) days from hospice enrollment to death were 36.37 ± 66.43 (19.00) days. The non-H group was significantly younger than both hospice subgroups. Both hospice subgroups were less likely to have esophageal bleeding and hemodialysis, but more likely to have diabetes, hypertension, and more hospital stays during the final month of life compared with the non-H group. All of these trends were significant (Table 1). We recorded the number of comorbidities as 0, 1, 2, 3, or >3. Comorbidities included diabetes, hypertension, stroke, liver cirrhosis, esophageal varices bleeding, portal hypertension, ascites, spontaneous bacterial peritonitis, hepatic encephalopathy, hepatorenal syndrome, hemodialysis, and chronic kidney diseases. Analysis showed no significant differences between non-H, short-H, and long-H groups among patients with comorbidities. However, we found a significantly higher proportion of patients with metastasis of advanced liver cancer in the long-H group than in the short-H and non-H groups (*p* < 0.001).

**Table 1:** Demographic characteristics of patients with liver cancer

Variable	Total	Non-H group	Short-H group	Long-H group	*p*-value	*p-*value for trend test
N (%)	3092(100 %)	2630(85.1 %)	325(10.5 %)	137(4.4 %)		
Gender					0.019	
female	876(28.3 %)	737(28.0 %)	86(26.5 %)	53(38.7 %)		0.072
male	2216(71.7 %)	1893(72.0 %)	239(73.5 %)	84(61.3 %)		0.072
Age	65.6 ± 13.37	65.9 ± 13.42	67.70 ± 12.42	67.50 ± 13.85	0.001	0.001
Survival (years)	1.43 ± 0.03	1.40 ± 0.04	1.52 ± 0.11	1.60 ± 0.17	0.212	0.162
Diabetes	446(14.4 %)	360(13.7 %)	54(16.6 %)	32(23.4 %)	0.004	0.001
Hypertension	2875(93.0 %)	2414(91.8 %)	324(99.7 %)	137(100 %)	<0.001	<0.001
Stroke	209(6.8 %)	169(6.4 %)	27(8.3 %)	13(9.5 %)	0.190	0.070
Liver cirrhosis	2108(68.2 %)	1794(68.2 %)	226(69.5 %)	88(64.2 %)	0.532	0.619
Esophageal varices bleeding	420(13.6 %)	381(14.5 %)	29(8.9 %)	10(7.3 %)	0.002	0.001
Portal hypertension	43(1.4 %)	37(1.4 %)	5(1.5 %)	1(0.7 %)	0.782	0.680
Ascites	908(29.4 %)	765(29.1 %)	102(31.4 %)	41(29.9 %)	0.685	0.518
SBP	23.8(7.7 %)	206(7.8 %)	24(7.4 %)	8(5.8 %)	0.678	0.405
Hepatic encephalopathy	766(24.8 %)	638(24.3 %)	92(28.3 %)	36(26.3 %)	0.257	0.189
Hepatorenal syndrome	99(3.2 %)	91(3.5 %)	4(1.2 %)	4(2.9 %)	0.076	0.138
Hemodialysis	147(4.8 %)	138(5.2 %)	6(1.8 %)	3(2.2 %)	0.001	0.005
CKD	183(5.9 %)	151(5.7 %)	21(6.5 %)	11(8.0 %)	0.431	0.245
Comorbidity of above						
No. of comorbidities = 0	141(4.6 %)	140(5.3 %)	1(0.3 %)	0	<0.001	<0.001
No. of comorbidities = 1	549(17.8 %)	473(18.0 %)	49(15.1 %)	27(19.7 %)	0.349	0.750
No. of comorbidities = 2	765(24.7 %)	630(24.0 %)	101(31.1 %)	34(24.8 %)	0.022	0.080
No. of comorbidities = 3	728(23.5 %)	613(23.3 %)	80(24.6 %)	35(25.5 %)	0.722	0.442
No. of comorbidities > 3	909(29.4 %)	774(29.4 %)	94(28.9 %)	41(29.9 %)	0.969	0.994
Metastasis	974(31.5 %)	781(29.7 %)	130(40.0 %)	63(46.0 %)	<0.001	<0.001
HBV	1079(34.9 %)	914(34.8 %)	123(37.8 %)	42(30.7 %)	0.308	0.877
HCV	832(26.9 %)	690(26.2 %)	100(30.8 %)	42(30.7 %)	0.129	0.061
CCI	3.69 ± 2.45	3.68 ± 2.40	3.68 ± 2.74	3.96 ± 2.80	0.284	0.284
SES						
LES	2080(67.3 %)	1764(67.1 %)	222(68.3 %)	94(68.6 %)	0.869	0.585
MES	800(25.9 %)	682(25.9 %)	83(25.5 %)	35(25.5 %)	0.996	0.746
HES	212(6.9 %)	184(7.0 %)	20(6.2 %)	8(5.8 %)	0.836	0.466
Urbanization level						
Urban	1513(48.9 %)	1292(49.1 %)	149(45.8 %)	72(52.6 %)	0.374	0.994
Suburban	1092(35.3 %)	937(35.6 %)	111(34.2 %)	44(32.1 %)	0.651	0.340
Rural	487(15.8 %)	401(15.2 %)	65(20.0 %)	21(15.3 %)	0.090	0.207
Teaching hospital^a^	1879(62.3 %)	1626(63.5 %)	169(53.5 %)	84(61.3 %)	0.003	0.021
Admission days^b^	13.7 ± 10.8	13.0 ± 10.9	16.5 ± 8.98	18.8 ± 11.3	<0.001	<0.001

^a^n = 3015

^b^Admission days: mean admission days in the last month

Abbreviations: Non-H group, patients who did not receive hospice care; Long-H group, patients who received hospice care for longer than 1 month; Short-H group, patients who received hospice care for a period shorter than 1 month; SBP, spontaneous bacterial peritonitis; CKD, chronic kidney disease; HBV, hepatitis B virus; HCV, hepatitis C virus; CCI, Charlson comorbidity index; SES, socioeconomic status; LES, low socioeconomic status; MES, moderate socioeconomic status; HES, high socioeconomic status

The main outcome measures in this study were the six QIs of EOL cancer care. Compared with patients in the long-H and short-H groups, a higher proportion of patients in the non-H group were admitted to an ICU (20.8 %) or received CPR (22.6 %) compared with patients in the long-H group (7.3 % and 6.6 %, respectively) and the short-H group (6.8 % and 2.8 %, respectively, *p*s < 0.001). A lower proportion of patients in the long-H group had more than one ER visit, compared with both those in the non-H and short-H groups (2.9 % vs. 4.6 %, 8.9 %, respectively, *p* = 0.003). A lower proportion of patients in the non-H group were admitted to a hospital more than once compared with those in the short-H and long-H groups (21.1 % vs. 31.7 %, 26.3 %, respectively, *p* < 0.001). Regarding the place of death, we found that fewer patients in the long-H group died in the hospice ward compared with patients in the short-H group (59.1 % and 68.0 %, respectively). We observed no significant difference regarding treatment with anticancer therapy during the final month of life among the three groups (*p* = 0.362). Further subanalysis revealed that more patients in the long-H group received radiotherapy compared with those in the short-H and non-H groups during this period (9.5 % vs. 7.7 %, 4.5 % respectively, *p* = 0.004) (Table 2).

**Table 2:** Comparison of quality indicators of end-of-life cancer care in the last month

Variables	Total	Non-H group	Short-H group	Long-H group	*p-*value	*p-*value for trend test
N (%)	3092(100 %)	2630(85.1 %)	325(10.5 %)	137(4.4 %)		
ICU admission	580(18.8 %)	548(20.8 %)	22(6.8 %)	10(7.3 %)	<0.001	<0.001
CPR	613(19.8 %)	595(22.6 %)	9(2.8 %)	9(6.6 %)	<0.001	<0.001
More than one ER visit	153(4.9 %)	120(4.6 %)	29(8.9 %)	4(2.9 %)	0.003	0.217
More than one admission(>1)	695(22.5 %)	556(21.1 %)	103(31.7 %)	36(26.3 %)	<0.001	<0.001
Death in acute care wards	1182(38.2 %)	1182(44.9 %)	0	0	-	-
Death in hospice ward	302(9.8 %)	0	221(68.0 %)	81(59.1 %)	-	-
Anti-cancer therapy						
Chemotherapy	61(2.0 %)	49(1.9 %)	9(2.8 %)	3(2.2 %)	0.459	0.406
TACE	128(4.1 %)	117(4.4 %)	7(2.2 %)	4(2.9 %)	0.117	0.074
HAIC	37(1.2 %)	30(1.1 %)	6(1.8 %)	1(0.7 %)	0.496	0.781
PEI	10(0.3 %)	10(0.4 %)	0	0	0.755	0.215
RFA	11(0.4 %)	11(0.4 %)	0	0	0.771	0.193
Radiotherapy	157(5.1 %)	119(4.5 %)	25(7.7 %)	13(9.5 %)	0.004	0.001
Ever anti-cancer therapy	348(11.3 %)	288(11.0 %)	41(12.6 %)	19(13.9 %)	0.362	0.183

Abbreviations: Non-H group, patients who did not receive hospice care; Long-H group, patients who received hospice care for longer than 1 month; Short-H group, patients who received hospice care for a period shorter than 1 month; ICU, intensive care unit; CPR, cardiopulmonary resuscitation; ER, emergency room; TACE, transarterial chemoembolizations; HAIC, hepatic arterial infusion chemotherapy; PEI, percutaneous ethanol injection; RFA: radiofrequency ablation

Multiple logistic regressions were performed to examine the QI risk factors. The long-H and short-H groups had a significantly lower risk of ICU admission (OR = 0.25, 95 % CI: 0.12–0.47 and OR = 0.26, 95 % CI: 0.16–0.40, respectively, *p* < 0.001) and receiving CPR (OR = 0.21, 95 % CI: 0.10–0.39 and OR = 0.09, 95 % CI: 0.04–0.17, respectively, *p* < 0.001) compared with the non-H group after adjustments. However, the short-H group had a higher risk of more than one ER visit, more than one admission, and death in the hospital (OR = 1.97, 95 % CI: 1.25–3.02, OR = 1.56, 95 % CI: 1.20–2.03, and OR = 2.42, 95 % CI: 1.86–3.17, respectively), whereas the long-H group had reduced risks (OR = 0.68, 95 % CI: 0.20–1.67, OR = 1.07, 95 % CI: 0.70–1.60, and OR = 1.29, 95 % CI: 0.87–1.94) compared with the non-H group (Table 3).

**Table 3:** Factors associated with quality indicators of end-of-life care among patients with liver cancer

Variable	ICU admission	CPR	Anti-cancer therapy	More than one ER visit	More than one admission	Death in hospital	One or more of above indicators
Long-H vs. non-H group	0.25(0.12-0.47) *p* < 0.001	0.21(0.10-0.39) *p* < 0.001	0.84(0.48-1.40) *p* = 0.525	0.68(0.20-1.67) *p* = 0.453	1.07(0.70-1.60) *p* = 0.762	1.29(0.87-1.94) *p* = 0.204	0.99(0.62-1.60) *p* = 0.954
Short-H vs. non-H group	0.26(0.16-0.40) *p* < 0.001	0.09(0.04-0.17) *p* < 0.001	1.00(0.68-1.42) *p* = 0.986	1.97(1.25-3.02) *p* = 0.003	1.56(1.20-2.03) *p* = 0.001	2.42(1.86-3.17) *p* < 0.001	1.56(1.13-2.18) *p* = 0.008
AUC	0.724(0.703-0.746)	0.703(0.681-0.725)	0.727(0.702-0.752)	0.736 (0.696-0.776)	0.711(0.691-0.731)	0.775(0.759-0.791)	0.833(0.816-0.850)
R^2^	0.159	0.142	0.124	0.110	0.142	0.295	0.418

Figures are odds ratios (confidence intervals) and associated *p* values

Abbreviations: Non-H group, patients who did not receive hospice care; Long-H group, patients who received hospice care for longer than 1 month; Short-H group, patients who received hospice care for a period shorter than 1 month; ICU, intensive care unit; CPR, cardiopulmonary resuscitation; AUC, area under the curve. All models were adjusted according to the significant variables shown in Tables 1 and 2. The full models are in Additional file 1

**Fig. 3:**
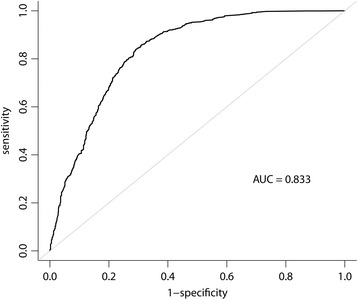
Area under the receiver operating characteristic curve (AUC). AUC was 0.833 for the prediction of receiving one or more indicators among patients with advanced liver cancer in their last month of life

We combined all the indicators and found that 2043 (66.1 %) patients had one or more of the six indicators. The short-H group had a higher risk of exhibiting one or more of the indicators compared with the non-H group (OR = 1.56, 95 % CI: 1.13–2.18, p = 0.008), whereas there was no significant difference between the long-H group and the non-H group (OR = 0.99, 95 % CI: 0.62–1.60, p = 0.954). All AUCs of these models exceeded 0.7, and the final model yielded the highest *R*^2^ (0.418) and AUC values (0.833) (Table 3 & Fig. 3). The complete models are listed in Additional file 1.

## Discussion

This study determined that hospice care, regardless of subgroup, reduced the likelihood of a patient being admitted to an ICU by approximately 75 % (long-H vs. non-H group, OR = 0.25; short-H vs. non-H group, OR = 0.26, *p* < 0.001) or receiving CPR by 80 %–90 % (long-H vs. non-H group, OR = 0.21; short-H vs. non-H group, 0.09, *p* < 0.001). However, the short-H group was 97 % more likely to have more than one ER visit (OR = 1.97, *p* = 0.003), and 56 % more likely to have more than one hospital admission (OR = 1.56, *p* = 0.001) compared with the non-H group. These three risks were lower for patients who received hospice care for more than 1 month. In addition, no differences in the three risks were observed between the long-H and non-H groups (OR = 0.68, p = 0.453; OR = 1.07, p = 0.762; and OR = 1.29, p = 0.204, respectively) and there were no differences regarding use of anticancer therapy among the three groups.

To the best of our knowledge, the results of this study are consistent with those of previous studies: that hospice care reduces the risk of admission to ICUs and use of CPR [8, 12–15]. Saito *et al.*, who investigated elderly hospice patients with non–small-cell lung cancer [15], and Gonsalves *et al.*, who examined advanced cancer patients in hospice care [14], have reported improvement in all six QIs compared with non-hospice patients. Obermeyer *et al.* reported that elderly hospice patients with cancer had fewer hospitalizations, ICU admissions, CPR treatments, and deaths in hospital than those not receiving hospice care; however, the researchers excluded patients receiving chemotherapy [13]. Barbera *et al.* reported fewer ER visits, ICU visits, and chemotherapy treatments in people receiving hospice care); however, they did not include the other three QIs [8]. One previous study indicated that Taiwanese cancer patients receiving hospital-based hospice care were significantly less likely to be intubated or require a mechanical ventilator [25].

Liver cancer is the third most common cause of death among cancers worldwide and has a high fatality rate (overall ratio of mortality to incidence: 0.93) [26]. It is the leading cause of cancer death in Taiwan (18.3 % in 2013) [27]. In this study, we found that patients with advanced liver cancer who were not receiving hospice care were likely to use ICU care (20.8 %) and receive CPR (22.6 %) in the last month of life, compared with patients who had received a short duration (6.8 % and 2.8 %, respectively) and long duration (7.3 % and 6.6 %, respectively) of hospice care. This may be because the most common causes of liver cancer death were liver-cancer-related or hepatic failure, followed by esophageal varices with bleeding, infections, and renal failure [28], which might incentivize patients to receive intensive care to control the progress or the complications of disease. However, patients who received hospice care required less ICU care and less CPR.

Hospice care is delivered mainly as an inpatient service and less as a home-based service in Taiwan. Hospice patients with advanced cancer might frequently visit ERs or be admitted to hospitals to receive services that relieve suffering. We found that patients in the short-H group had more ER visits and hospitalizations than did those in the non-H and long-H groups. There are several possible reasons for this pattern. First, the short-H group might have required more medical assistance for symptom control; second, the patients’ symptoms or their informal hospice caregivers’ skills [29] might have been improved after long-term hospice care; third, the communication between patients/their families and nurses might have improved after long-term hospice care [30]. Dudgeon *et al.*, who enrolled patients with all types of cancer in a study assessing a palliative care integration strategy, reported fewer ER visits and hospital admissions but no significant decreases in the percentage of deaths in hospital; however, they did not examine the other three QIs [12]. A previous study investigating why Taiwanese cancer patients preferred to stay in hospital suggested that a more effective referral system be established and that home-based hospice services be provided [31]. Education could have a significant effect on EOL care [32]. Our findings suggest that EOL cancer care in Taiwan might be optimized if health care policies promoted early hospice referral programs (longer than 1 month) and education for preparing patients and families for death.

Despite increases in the use of palliative and hospice care in elderly patients with terminal illness, ethnic disparities persist [33]. Therefore, hospice care teams who understand cultural differences between Chinese and Western societies might improve EOL quality care. In traditional Chinese culture, death is a sensitive issue and people avoid mentioning it, as to do so is considered sacrilegious [34]. This might explain why there was more than one admission to an acute care hospital in the last month of life for patients in the non-H group (21.1 %), and more than one hospice ward admission in the last month of life for patients in the short-H and long-H groups (31.7 % and 26.3 %, respectively). “The fallen leaves can return to their roots” is an important traditional Chinese religious concept [35]. Therefore, dying at home has a special meaning for patients and their families in Taiwan. Dying patients in Taiwan were commonly discharged “against medical advice” from the hospital and often with artificial respiratory support (e.g., nasal cannula) to allow their families to keep the patient “alive” long enough to enable them to die at home [9]. Accordingly, if the date of discharge for the last admission was the same as the date of death [22], the patients were recognized as dying in the acute care ward or in the hospice ward (if they had been admitted to a hospice).

Although many experts recommend a hospice stay of at least 3 months to provide standard hospice services [36], the average length of hospice stay is about 1 month in the United States [37]. One previous study reported that late hospice referral could increase the risk of a major depressive disorder during the first year of bereavement [38], result in lower reported satisfaction with hospice services by family members [39], and be related to the quality of patient care received [40]. In a multicenter survey, only 1.5 % of families assessed the timing of home-based hospice referral as early and 42.0 % assessed it as late [41]. Patients who are referred to hospice care early in their disease process can benefit more easily from this care, which may improve patients’ quality of life, patient and family satisfaction, and the cost effectiveness of treatment [42]. The barriers to early referral are related to physician and patient/family attitudes as well as the reimbursement structure itself. Physician-related barriers include a reluctance to discuss hospice care because of fears about the patient’s/family’s reaction, difficulty in survival prediction, feelings of professional failure, and loss of control [43–46]. Patient-related barriers include denial of health status, a desire to exhaust all treatment options, a negative perception of hospice care, and patient demographics [42,47]. Another challenging issue is the prediction of survival for patients with advanced cancer. To date, clinician prediction of survival is one of the most practical and commonly used temporal approaches for prognosis estimation in spite of its tendency to overestimate survival time [48,49]. Clinicians specialized in palliative care need to be proficient at prognosis to provide the best EOL care for their patients. Accurate survival prediction allows patients and their families to develop insight into their dying and thereby set realistic priorities and expectations of care.

### Limitations

This study has some limitations. One limitation is the possibility of misclassification bias because of the inaccuracy of some of the variables used, including the calculation of the comorbidity score. Another limitation is the possibility of selection bias, which might have occurred because the participant allocation was not randomized. Moreover, the rate of cirrhosis and its complications, such as esophageal bleeding and ascites, might have been underestimated because our data were obtained from claims data and not medical records. Furthermore, this reliance on claims data made it impossible to collect information on clinical symptoms and signs, patient or family preferences, or do-not-resuscitate designations. In addition, patient or family decision making may have influenced some of the outcomes.

## Conclusion

There was no significant difference in survival probability between the hospice groups. Both hospice groups were less likely to require ICU admissions or CPR during the final month of life. However, the short-H group exhibited a higher risk of more than one ER visit, and more than one hospital admission, compared with the non-H and long-H groups. We conclude that EOL cancer care in Taiwan might be improved by policies encouraging early hospice referral programs (longer than 1 month) and more realistic education that prepares patients and their families for imminent death.

## Additional file

Additional file 1:
**Factors associated with quality indicators of end-of-life care among patients with liver cancer.** Figures are odds ratios (confidence intervals) and associated *p* values. Abbreviations: Non-H group, patients who did not receive hospice care; Long-H group, patients who received hospice care for longer than 1 month; Short-H group, patients who received hospice care for a period shorter than 1 month; ICU, intensive care unit; CPR, cardiopulmonary resuscitation; ER, emergency room; HBV, hepatitis B virus; HCV, hepatitis C virus; SBP, spontaneous bacterial peritonitis; CKD, chronic kidney disease; LES, low socioeconomic status; MES, moderate socioeconomic status; HES, high socioeconomic status; PPV, positive predictive value; NPV, negative predictive value; AUC, area under the curve; HL, Hosmer–Lemeshow. (DOCX 18 kb)

## Abbreviations

AUC: area under the receiver operating characteristic curve
CCI: Charlson comorbidity index
CIC: catastrophic illness certificate
CKD: chronic kidney disease
CPR: cardiopulmonary resuscitation
DNR: Do not resuscitate
EOL: end-of-life
ER: emergency room
HAIC: hepatic arterial infusion chemotherapy
HBV: hepatitis B virus
HCV: hepatitis C virus
ICD-9-CM: International Classification of Diseases, Ninth Revision, Clinical Modification
ICU: intensive care unit
NHI: National Health Insurance
NHIRD: National Health Insurance Research Database
PEI: percutaneous ethanol injection
QI: quality indicator
RFA: radiofrequency ablation
SES: socioeconomic status
TACE: transarterial chemoembolization
