# Supplementary material for: Effect of hospice care on quality indicators of end-of-life care among patients with liver cancer: a national longitudinal population-based study in Taiwan 2000–2011
Source: BMC Palliat Care. 2015 Aug 19;14:39. doi: 10.1186/s12904-015-0036-9 (PMC4545784; doi:10.1186/s12904-015-0036-9)
Supplement: Additional file 1: — Factors associated with quality indicators of end-of-life care among patients with liver cancer. Figures are odds ratios (confidence intervals) and associated p values. Abbreviations: Non-H group, patients who did not receive hospice care; Long-H group, patients who received hospice care for longer than 1 month; Short-H group, patients who received hospice care for a period shorter than 1 month; ICU, intensive care unit; CPR, cardiopulmonary resuscitation; ER, emergency room; HBV, hepatitis B virus; HCV, hepatitis C virus; SBP, spontaneous bacterial peritonitis; CKD, chronic kidney disease; LES, low socioeconomic status; MES, moderate socioeconomic status; HES, high socioeconomic status; PPV, positive predictive value; NPV, negative predictive value; AUC, area under the curve; HL, Hosmer–Lemeshow. (DOCX 18 kb) [file 12904_2015_36_MOESM1_ESM.docx]

**Additional File 1.**

**Factors associated with quality indicators of end-of-life care among patients with liver cancer**

| Variable | ICU admission | CPR | Anti-cancer therapy | More than  one ER visit | More than  one admission | Death in hospital | One or more of above  indicators |
| --- | --- | --- | --- | --- | --- | --- | --- |
| Long-H vs. non-H group | 0.25(0.12-0.47)  *p*<0.001 | 0.21(0.10-0.39)  *p*<0.001 | - | 0.68(0.20-1.67)  *p*=0.453 | 1.07(0.70-1.60)  *p*=0.762 | 1.29(0.87-1.94)  *p*=0.204 | 0.99(0.62-1.60)  *p*=0.954 |
| Short-H vs. non-H group | 0.26(0.16-0.40)  *p*<0.001 | 0.09(0.04-0.17)  *p*<0.001 | - | 1.97(1.25-3.02)  *p*=0.003 | 1.56(1.20-2.03)  *p*=0.001 | 2.42(1.86-3.17)  *p*<0.001 | 1.56(1.13-2.18)  *p*=0.008 |
| Age (per 5 years) | - | - | 0.88(0.84-0.92)  *p*< 0.001 | - | - |  |  |
| Age 30-65 year | - | - | - | - | 1.24(1.03-1.50)  *p*=0.026 | - |  |
| Male vs. female | - | - | - | 1.79(1.17-2.85)  *p*=0.010 | - | - |  |
| HBV | - | 0.73(0.59-0.89)  *p*=0.003 | - | 1.78(1.25-2.53)  *p*=0.001 | 1.63(1.33-1.98)  *p*<0.001 | 1.35(1.13-1.62)  *p*=0.001 | 1.49(1.21-1.85)  *p*<0.001 |
| HCV | - | - | 1.59(1.22-2.07)  *p*=0.001 | 2.73(1.93-3.88)  *p*<0.001 | 1.35(1.09-1.66)  *p*=0.005 | 1.33(1.10-1.61)  *p*=0.004 | 1.39(1.11-1.74)  *p*=0.004 |
| SBP | - | - | 0.50(0.29-0.80)  *p*=0.006 | 2.10(1.30-3.28)  *p*=0.002 | - | - |  |
| Stroke | 1.54(1.08-2.17)  *p*=0.015 | - | - | - | - | - | 1.52(1.05-2.22)  *p*=0.030 |
| Hypertension | - | - | 2.11(1.16-4.22)  *p*=0.022 | - | - | - | 4.62(2.50-8.85)  *p*<0.001 |
| Diabetes | - | - | - | - | 1.43(1.13-1.80)  *p*=0.003 | - |  |
| EVB | 1.80(1.40-2.31)  *p*<0.001 | 1.50(1.16-1.93)  *p*=0.002 | 0.69(0.48-0.97)  *p*=0.037 |  | - | - |  |
| Cirrhosis | 1.37(1.09-1.73)  *p*=0.007 | 1.47(1.17-1.85)  *p*=0.001 | - |  | 1.35(1.06-1.71)  *p*=0.015 | 1.43(1.18-1.73)  *p*<0.011 | 1.70(1.36-2.14)  *p*<0.011 |
| Ascites | - | 0.78(0.63-0.97)  *p*=0.025 | - | 1.55(1.09-2.20)  *p*=0.014 | 1.24(1.02-1.51)  *p*=0.030 | - |  |
| Hepatic encephalopathy | - | - | 0.59(0.44-0.79)  *p*<0.001 | - | 1.67(1.37-2.03)  *p*<0.001 | - | 1.46(1.16-1.85)  *p*=0.002 |
| CKD | 0.53(0.33-0.85)  *p*=0.010 | 0.61(0.38-0.95)  *p*=0.035 | - | - | - | - |  |
| Hemodialysis | 6.97(4.57-10.8)  *p*<0.001 | 4.69(3.12-7.13)  *p*<0.001 | - | - | - | 1.58(1.09-2.29)  *p*=0.016 |  |
| Urban | - | - | - | - | - | 1.63(1.37-1.95)  *p*<0.001 |  |
| Suburban | - | - | 1.41(1.11-1.78)  *p*=0.005 | - | 1.28(1.06-1.53)  *p* =0.010 | - |  |
| Rural | - | - | - | - | - | 0.73(0.57-0.94)  *p* =0.014 | 0.63(0.49-0.82)  *p*<0.001 |
| LES | - | - | - | 0.47(0.33-0.65)  *p*<0.001 | - | 0.41(0.34-0.49)  *p*<0.001 |  |
| MES |  |  |  |  |  |  | 1.93(1.54-2.43)  *p*<0.001 |
| HES | - | - | - | - | - | 0.64(0.45-0.91)  *p* =0.013 |  |
| Admission days | 1.05(1.04-1.06)  *p*<0.001 | 1.04(1.03-1.05)  *p*<0.001 | 1.07(1.05-1.08)  *p*<0.001 | - | 1.05(1.04-1.05)  *p*<0.001 | 1.08(1.07-1.09)  *p*<0.001 | 1.12(1.11-1.34)  *p*<0.001 |
| Sensitivity | 0.71(0.68-0.75) | 0.71(0.67-0.74) | 0.79(0.74-0.83) | 0.67(0.59-0.74) | 0.68(0.64-0.71) | 0.73(0.70-0.75) | 0.79(0.77-0.81) |
| Specificity | 0.62(0.60-0.64) | 0.58(0.56-0.60) | 0.57(0.56-0.59) | 0.66(0.64-0.68) | 0.63(0.61-0.65) | 0.68(0.66-0.70) | 0.74(0.72-0.77) |
| PPV | 0.30(0.28-0.33) | 0.30(0.27-0.32) | 0.19(0.17-0.21) | 0.09(0.08-0.11) | 0.35(0.32-0.37) | 0.68(0.65-0.70) | 0.86(0.84-0.87) |
| NPV | 0.90(0.89-0.92) | 0.89(0.87-0.90) | 0.96(0.94-0.97) | 0.97(0.97-0.98) | 0.87(0.85-0.89) | 0.73(0.71-0.75) | 0.65(0.62-0.67) |
| AUC | 0.724  (0.703-0.746) | 0.703  (0.681-0.725) | 0.727  (0.702-0.752) | 0.736  (0.696-0.776) | 0.711  (0.691-0.731) | 0.775  (0.759-0.791) | 0.833  (0.816-0.850) |
| R^2^ | 0.159 | 0.142 | 0.124 | 0.110 | 0.142 | 0.295 | 0.418 |
| HL test *p* value | <0.001 | <0.001 | 0.110 | 0.244 | <0.001 | <0.001 | <0.001 |

Figures are odds ratios (confidence intervals) and associated *p* values.

Abbreviations: Non-H group, patients who did not receive hospice care; Long-H group, patients who received hospice care for longer than 1 month; Short-H group, patients who received hospice care for a period shorter than 1 month; ICU, intensive care unit; CPR, cardiopulmonary resuscitation; ER, emergency room; HBV, hepatitis B virus; HCV, hepatitis C virus; SBP, spontaneous bacterial peritonitis; CKD, chronic kidney disease; LES, low socioeconomic status; MES, moderate socioeconomic status; HES, high socioeconomic status; PPV, positive predictive value; NPV, negative predictive value; AUC, area under the curve; HL, Hosmer–Lemeshow.
